# Di-μ-chlorido-bis­{chlorido[4-nitro-*N*-(pyridin-2-yl­methyl­idene-κ*N*)aniline-κ*N*]mercury(II)}

**DOI:** 10.1107/S1600536811004703

**Published:** 2011-02-12

**Authors:** Sadegh Salehzadeh, Saeed Dehghanpour, Mehdi Khalaj, Mohammad Rahimishakiba

**Affiliations:** a Faculty of Chemistry, Bu-Ali Sina University, Hamedan, Iran; b Department of Chemistry, Alzahra University, Vanak, Tehran, Iran; c Department of Chemistry, Islamic Azad University, Buinzahra Branch, Buinzahra, Qazvin, Iran

## Abstract

In the centrosymmetric dinuclear title complex, [Hg_2_Cl_4_(C_12_H_9_N_3_O_2_)_2_], the Hg^II^ ion is in a distorted square-pyramidal coordination environment formed by the N atoms of the diimine ligand, two bridging Cl atoms and one terminal Cl atom. One of the bridging Hg—Cl bonds is significantly longer than the other.

## Related literature

For background to diimine complexes and related structures, see: Dehghanpour *et al.* (2007 ▶); Mahmoudi *et al.* (2009 ▶).
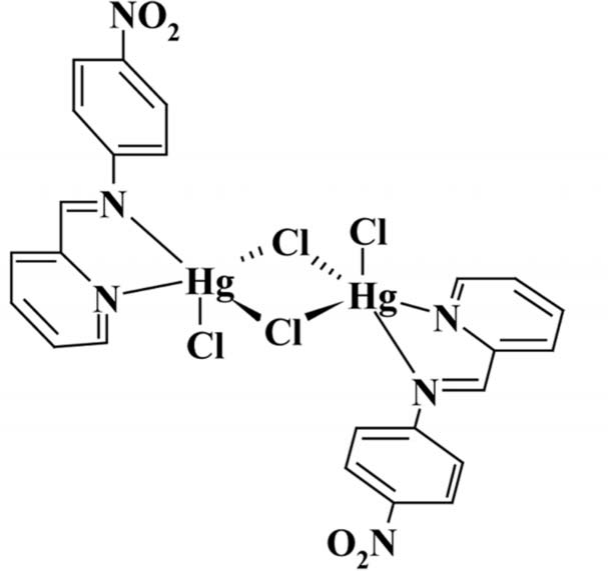


## Experimental

### 

#### Crystal data

[Hg_2_Cl_4_(C_12_H_9_N_3_O_2_)_2_]
*M* *_r_* = 997.42
Monoclinic, 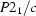

*a* = 8.9731 (2) Å
*b* = 7.8439 (3) Å
*c* = 20.1403 (7) Å
β = 98.155 (2)°
*V* = 1403.22 (8) Å^3^
*Z* = 2
Mo *K*α radiation
μ = 11.35 mm^−1^
*T* = 150 K
0.18 × 0.16 × 0.14 mm

#### Data collection

Nonius KappaCCD diffractometer
Absorption correction: multi-scan (*SORTAV*; Blessing, 1995 ▶) *T*_min_ = 0.115, *T*_max_ = 0.222
11428 measured reflections
3197 independent reflections
2639 reflections with *I* > 2σ(*I*)
*R*_int_ = 0.055

#### Refinement

*R*[*F* ^2^ > 2σ(*F* ^2^)] = 0.028
*wR*(*F* ^2^) = 0.064
*S* = 1.06
3197 reflections
181 parameters
H-atom parameters constrained
Δρ_max_ = 0.94 e Å^−3^
Δρ_min_ = −1.56 e Å^−3^

### 

Data collection: *COLLECT* (Nonius, 2002 ▶); cell refinement: *DENZO-SMN* (Otwinowski & Minor, 1997 ▶); data reduction: *DENZO-SMN*; program(s) used to solve structure: *SIR92* (Altomare *et al.*, 1994 ▶); program(s) used to refine structure: *SHELXTL* (Sheldrick, 2008 ▶); molecular graphics: *PLATON* (Spek, 2009 ▶); software used to prepare material for publication: *SHELXTL*.

## Supplementary Material

Crystal structure: contains datablocks I, global. DOI: 10.1107/S1600536811004703/gk2347sup1.cif

Structure factors: contains datablocks I. DOI: 10.1107/S1600536811004703/gk2347Isup2.hkl

Additional supplementary materials:  crystallographic information; 3D view; checkCIF report

## Figures and Tables

**Table 1:** Selected bond lengths (Å)

Hg1—N1	2.323 (4)
Hg1—Cl1	2.3940 (11)
Hg1—N2	2.442 (4)
Hg1—Cl2	2.5161 (12)
Hg1—Cl2^i^	2.8068 (11)

Symmetry code: (i) 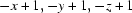.

**Fig. 1.**
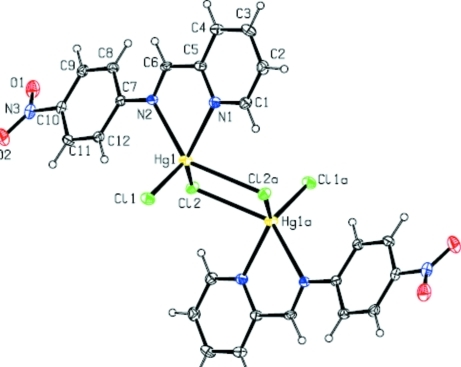
A view of the structure of the title complex, with displacement ellipsoids drawn at the 50% probability level. Symmetry code: (a) -x + 1, -y + 1, -z + 1.

## supplementary crystallographic information

### Comment

In our ongoing studies on the synthesis, structural and spectroscopic characterization of transition metal complexes with diimine ligands (Dehghanpour *et al.,* 2007; Mahmoudi *et al.,* 2009), we report herein the crystal structure of the title complex. The title compound was prepared by the reaction of HgCl_2_ with (4-nitrophenyl)pyridin-2-ylmethyleneamine.

The molecluar structure of the title complex is shown in Fig. 1. The unique Hg^II^ ion in is in a distorted square pyramidal coordination environment formed by a bis-chelating ligand, two bridging Cl atoms and one terminal Cl atom.

### Experimental

The title complex was prepared by the reaction of HgCl_2_ (22.7 mg, 0.1 mmol) and (4-nitrophenyl)pyridin-2-ylmethyleneamine (27.2 mg, 0.1mmol) in 15 ml acetonitrile at room temperature. The solution was then concentrated under vacuum, and diffusion of diethyl ether vapor into the concentrated solution gave yellow crystals of the title compound in 60% yield.

### Refinement

The H- atom positions were calculated and refined in a riding model approximatiom with *U*_iso_(H) = 1.2*U*_eq_(C).

### Crystal data

**Table:**

[Hg_2_Cl_4_(C_12_H_9_N_3_O_2_)_2_]	*F*(000) = 928
*M**_r_* = 997.42	*D*_x_ = 2.361 Mg m^−^^3^
Monoclinic, *P*2_1_/*c*	Mo *K*α radiation, λ = 0.71073 Å
Hall symbol: -P 2ybc	Cell parameters from 6448 reflections
*a* = 8.9731 (2) Å	θ = 2.6–27.5°
*b* = 7.8439 (3) Å	µ = 11.35 mm^−^^1^
*c* = 20.1403 (7) Å	*T* = 150 K
β = 98.155 (2)°	Block, colourless
*V* = 1403.22 (8) Å^3^	0.18 × 0.16 × 0.14 mm
*Z* = 2	

### Data collection

**Table:**

Nonius KappaCCD diffractometer	3197 independent reflections
Radiation source: fine-focus sealed tube	2639 reflections with *I* > 2σ(*I*)
graphite	*R*_int_ = 0.055
Detector resolution: 9 pixels mm^-1^	θ_max_ = 27.6°, θ_min_ = 2.8°
φ scans and ω scans with κ offsets	*h* = −11→11
Absorption correction: multi-scan (*SORTAV*; Blessing, 1995)	*k* = −9→10
*T*_min_ = 0.115, *T*_max_ = 0.222	*l* = −25→26
11428 measured reflections	

### Refinement

**Table:**

Refinement on *F*^2^	Primary atom site location: structure-invariant direct methods
Least-squares matrix: full	Secondary atom site location: difference Fourier map
*R*[*F*^2^ > 2σ(*F*^2^)] = 0.028	Hydrogen site location: inferred from neighbouring sites
*wR*(*F*^2^) = 0.064	H-atom parameters constrained
*S* = 1.06	*w* = 1/[σ^2^(*F*_o_^2^) + (0.0234*P*)^2^ + 1.6835*P*] where *P* = (*F*_o_^2^ + 2*F*_c_^2^)/3
3197 reflections	(Δ/σ)_max_ = 0.001
181 parameters	Δρ_max_ = 0.94 e Å^−^^3^
0 restraints	Δρ_min_ = −1.56 e Å^−^^3^

### Special details

**Table:**

Geometry. All e.s.d.'s (except the e.s.d. in the dihedral angle between two l.s. planes) are estimated using the full covariance matrix. The cell e.s.d.'s are taken into account individually in the estimation of e.s.d.'s in distances, angles and torsion angles; correlations between e.s.d.'s in cell parameters are only used when they are defined by crystal symmetry. An approximate (isotropic) treatment of cell e.s.d.'s is used for estimating e.s.d.'s involving l.s. planes.
Refinement. Refinement of *F*^2^ against ALL reflections. The weighted *R*-factor *wR* and goodness of fit *S* are based on *F*^2^, conventional *R*-factors *R* are based on *F*, with *F* set to zero for negative *F*^2^. The threshold expression of *F*^2^ > σ(*F*^2^) is used only for calculating *R*-factors(gt) *etc*. and is not relevant to the choice of reflections for refinement. *R*-factors based on *F*^2^ are statistically about twice as large as those based on *F*, and *R*- factors based on ALL data will be even larger.

### Fractional atomic coordinates and isotropic or equivalent isotropic displacement parameters (Å^2^)

**Table:**

	*x*	*y*	*z*	*U*_iso_*/*U*_eq_	
Hg1	0.641245 (19)	0.62356 (2)	0.448116 (9)	0.02542 (8)	
Cl1	0.83299 (13)	0.45830 (16)	0.40698 (7)	0.0314 (3)	
Cl2	0.60508 (13)	0.60053 (14)	0.56941 (6)	0.0270 (3)	
O1	1.0314 (4)	1.2850 (5)	0.72381 (19)	0.0428 (9)	
O2	1.1963 (4)	1.0918 (5)	0.7078 (2)	0.0440 (10)	
N1	0.5013 (4)	0.7782 (5)	0.36266 (19)	0.0238 (8)	
N2	0.6929 (4)	0.9273 (5)	0.4652 (2)	0.0216 (8)	
N3	1.0753 (5)	1.1652 (5)	0.6919 (2)	0.0319 (10)	
C1	0.4077 (5)	0.7090 (6)	0.3123 (2)	0.0265 (10)	
H1A	0.3985	0.5884	0.3101	0.032*	
C2	0.3234 (5)	0.8064 (7)	0.2632 (3)	0.0308 (11)	
H2A	0.2589	0.7531	0.2277	0.037*	
C3	0.3344 (5)	0.9808 (7)	0.2663 (3)	0.0312 (11)	
H3A	0.2769	1.0502	0.2335	0.037*	
C4	0.4315 (5)	1.0539 (6)	0.3187 (2)	0.0275 (10)	
H4A	0.4404	1.1744	0.3222	0.033*	
C5	0.5152 (5)	0.9497 (6)	0.3657 (2)	0.0212 (9)	
C6	0.6209 (5)	1.0210 (6)	0.4202 (3)	0.0228 (10)	
H6A	0.6364	1.1408	0.4221	0.027*	
C7	0.7901 (5)	0.9952 (6)	0.5209 (2)	0.0206 (9)	
C8	0.7776 (5)	1.1611 (6)	0.5433 (2)	0.0239 (10)	
H8A	0.7039	1.2351	0.5203	0.029*	
C9	0.8717 (5)	1.2189 (6)	0.5988 (2)	0.0265 (10)	
H9A	0.8641	1.3324	0.6145	0.032*	
C10	0.9778 (5)	1.1072 (6)	0.6314 (2)	0.0242 (10)	
C11	0.9934 (5)	0.9425 (6)	0.6092 (2)	0.0257 (10)	
H11A	1.0688	0.8696	0.6316	0.031*	
C12	0.8981 (5)	0.8855 (6)	0.5542 (2)	0.0241 (10)	
H12A	0.9058	0.7717	0.5388	0.029*	

### Atomic displacement parameters (Å^2^)

**Table:**

	*U*^11^	*U*^22^	*U*^33^	*U*^12^	*U*^13^	*U*^23^
Hg1	0.03088 (12)	0.01758 (11)	0.02789 (12)	0.00384 (7)	0.00446 (8)	0.00096 (8)
Cl1	0.0311 (6)	0.0273 (6)	0.0363 (7)	0.0085 (5)	0.0069 (5)	−0.0012 (5)
Cl2	0.0332 (6)	0.0244 (6)	0.0233 (6)	−0.0052 (4)	0.0035 (5)	−0.0015 (5)
O1	0.037 (2)	0.046 (2)	0.043 (2)	−0.0073 (18)	0.0020 (17)	−0.017 (2)
O2	0.0277 (19)	0.055 (3)	0.045 (2)	0.0031 (17)	−0.0075 (16)	−0.0044 (19)
N1	0.0249 (19)	0.022 (2)	0.025 (2)	0.0021 (16)	0.0057 (16)	0.0018 (17)
N2	0.0210 (18)	0.0192 (19)	0.025 (2)	0.0007 (15)	0.0039 (15)	0.0047 (16)
N3	0.026 (2)	0.037 (3)	0.033 (3)	−0.0090 (18)	0.0047 (18)	−0.003 (2)
C1	0.029 (2)	0.026 (3)	0.024 (3)	−0.003 (2)	0.0052 (19)	0.001 (2)
C2	0.029 (2)	0.035 (3)	0.026 (3)	−0.004 (2)	−0.004 (2)	−0.004 (2)
C3	0.033 (3)	0.036 (3)	0.023 (3)	0.012 (2)	0.000 (2)	0.006 (2)
C4	0.035 (3)	0.021 (2)	0.026 (3)	0.004 (2)	0.003 (2)	0.001 (2)
C5	0.026 (2)	0.021 (2)	0.017 (2)	0.0034 (19)	0.0048 (18)	0.0020 (19)
C6	0.027 (2)	0.013 (2)	0.029 (3)	0.0024 (18)	0.0064 (19)	−0.001 (2)
C7	0.019 (2)	0.019 (2)	0.024 (3)	0.0006 (17)	0.0067 (18)	0.0006 (19)
C8	0.025 (2)	0.022 (2)	0.024 (3)	0.0018 (18)	0.0044 (19)	0.0039 (19)
C9	0.025 (2)	0.020 (2)	0.035 (3)	−0.0029 (19)	0.009 (2)	−0.006 (2)
C10	0.020 (2)	0.026 (3)	0.027 (3)	−0.0062 (18)	0.0036 (18)	−0.002 (2)
C11	0.024 (2)	0.023 (2)	0.030 (3)	0.004 (2)	0.0027 (19)	0.004 (2)
C12	0.022 (2)	0.020 (2)	0.031 (3)	−0.0005 (18)	0.0053 (19)	−0.001 (2)

### Geometric parameters (Å, °)

**Table:**

Hg1—N1	2.323 (4)	C3—C4	1.392 (7)
Hg1—Cl1	2.3940 (11)	C3—H3A	0.9500
Hg1—N2	2.442 (4)	C4—C5	1.388 (6)
Hg1—Cl2	2.5161 (12)	C4—H4A	0.9500
Hg1—Cl2^i^	2.8068 (11)	C5—C6	1.457 (6)
Cl2—Hg1^i^	2.8068 (11)	C6—H6A	0.9500
O1—N3	1.233 (5)	C7—C8	1.387 (6)
O2—N3	1.231 (5)	C7—C12	1.394 (6)
N1—C1	1.337 (6)	C8—C9	1.380 (7)
N1—C5	1.352 (6)	C8—H8A	0.9500
N2—C6	1.272 (6)	C9—C10	1.388 (7)
N2—C7	1.423 (6)	C9—H9A	0.9500
N3—C10	1.468 (6)	C10—C11	1.381 (6)
C1—C2	1.386 (7)	C11—C12	1.375 (7)
C1—H1A	0.9500	C11—H11A	0.9500
C2—C3	1.372 (7)	C12—H12A	0.9500
C2—H2A	0.9500		
			
N1—Hg1—Cl1	111.46 (10)	C4—C3—H3A	120.7
N1—Hg1—N2	70.57 (13)	C5—C4—C3	119.6 (5)
Cl1—Hg1—N2	116.47 (9)	C5—C4—H4A	120.2
N1—Hg1—Cl2	128.76 (9)	C3—C4—H4A	120.2
Cl1—Hg1—Cl2	119.68 (4)	N1—C5—C4	121.2 (4)
N2—Hg1—Cl2	88.91 (9)	N1—C5—C6	117.6 (4)
N1—Hg1—Cl2^i^	84.28 (9)	C4—C5—C6	121.3 (4)
Cl1—Hg1—Cl2^i^	102.07 (4)	N2—C6—C5	121.8 (4)
N2—Hg1—Cl2^i^	139.38 (9)	N2—C6—H6A	119.1
Cl2—Hg1—Cl2^i^	82.50 (4)	C5—C6—H6A	119.1
Hg1—Cl2—Hg1^i^	97.50 (4)	C8—C7—C12	120.4 (4)
C1—N1—C5	118.8 (4)	C8—C7—N2	122.6 (4)
C1—N1—Hg1	124.5 (3)	C12—C7—N2	117.0 (4)
C5—N1—Hg1	116.7 (3)	C9—C8—C7	120.2 (4)
C6—N2—C7	122.6 (4)	C9—C8—H8A	119.9
C6—N2—Hg1	113.3 (3)	C7—C8—H8A	119.9
C7—N2—Hg1	124.0 (3)	C8—C9—C10	118.4 (4)
O2—N3—O1	123.8 (4)	C8—C9—H9A	120.8
O2—N3—C10	118.1 (4)	C10—C9—H9A	120.8
O1—N3—C10	118.1 (4)	C11—C10—C9	122.2 (4)
N1—C1—C2	122.6 (5)	C11—C10—N3	118.9 (4)
N1—C1—H1A	118.7	C9—C10—N3	119.0 (4)
C2—C1—H1A	118.7	C12—C11—C10	119.0 (4)
C3—C2—C1	119.2 (5)	C12—C11—H11A	120.5
C3—C2—H2A	120.4	C10—C11—H11A	120.5
C1—C2—H2A	120.4	C11—C12—C7	119.8 (4)
C2—C3—C4	118.6 (4)	C11—C12—H12A	120.1
C2—C3—H3A	120.7	C7—C12—H12A	120.1
			
N1—Hg1—Cl2—Hg1^i^	76.43 (12)	C1—N1—C5—C6	−178.4 (4)
Cl1—Hg1—Cl2—Hg1^i^	−99.66 (5)	Hg1—N1—C5—C6	2.7 (5)
N2—Hg1—Cl2—Hg1^i^	140.20 (9)	C3—C4—C5—N1	−1.8 (7)
Cl2^i^—Hg1—Cl2—Hg1^i^	0.0	C3—C4—C5—C6	178.4 (4)
Cl1—Hg1—N1—C1	68.2 (4)	C7—N2—C6—C5	−176.3 (4)
N2—Hg1—N1—C1	179.9 (4)	Hg1—N2—C6—C5	1.9 (5)
Cl2—Hg1—N1—C1	−108.1 (3)	N1—C5—C6—N2	−3.2 (6)
Cl2^i^—Hg1—N1—C1	−32.5 (3)	C4—C5—C6—N2	176.7 (4)
Cl1—Hg1—N1—C5	−112.9 (3)	C6—N2—C7—C8	22.2 (7)
N2—Hg1—N1—C5	−1.3 (3)	Hg1—N2—C7—C8	−155.9 (3)
Cl2—Hg1—N1—C5	70.7 (3)	C6—N2—C7—C12	−159.7 (4)
Cl2^i^—Hg1—N1—C5	146.3 (3)	Hg1—N2—C7—C12	22.3 (5)
N1—Hg1—N2—C6	−0.3 (3)	C12—C7—C8—C9	−0.2 (7)
Cl1—Hg1—N2—C6	104.6 (3)	N2—C7—C8—C9	177.9 (4)
Cl2—Hg1—N2—C6	−132.5 (3)	C7—C8—C9—C10	−0.2 (7)
Cl2^i^—Hg1—N2—C6	−55.3 (4)	C8—C9—C10—C11	1.2 (7)
N1—Hg1—N2—C7	177.8 (3)	C8—C9—C10—N3	−177.9 (4)
Cl1—Hg1—N2—C7	−77.2 (3)	O2—N3—C10—C11	23.7 (6)
Cl2—Hg1—N2—C7	45.7 (3)	O1—N3—C10—C11	−156.4 (4)
Cl2^i^—Hg1—N2—C7	122.8 (3)	O2—N3—C10—C9	−157.1 (4)
C5—N1—C1—C2	−0.4 (7)	O1—N3—C10—C9	22.7 (6)
Hg1—N1—C1—C2	178.4 (3)	C9—C10—C11—C12	−1.8 (7)
N1—C1—C2—C3	−0.8 (7)	N3—C10—C11—C12	177.3 (4)
C1—C2—C3—C4	0.7 (7)	C10—C11—C12—C7	1.4 (7)
C2—C3—C4—C5	0.5 (7)	C8—C7—C12—C11	−0.5 (7)
C1—N1—C5—C4	1.7 (6)	N2—C7—C12—C11	−178.7 (4)
Hg1—N1—C5—C4	−177.2 (3)		

Symmetry codes: (i) −*x*+1, −*y*+1, −*z*+1.
